# Use of Monoclonal Antibodies in the Sensitive Detection and Neutralization of Botulinum Neurotoxin Serotype B

**DOI:** 10.3390/toxins7124863

**Published:** 2015-11-27

**Authors:** Luisa W. Cheng, Thomas D. Henderson, Tina I. Lam, Larry H. Stanker

**Affiliations:** 1 Foodborne Toxin Detection and Prevention Research Unit, Western Regional Research Center, Agricultural Research Service, United States Department of Agriculture, Albany, CA 94710, USA; thomas.henderson@ars.usda.gov (T.D.H.); larry.stanker@ars.usda.gov (L.H.S.); 2 Gilead Sciences, Inc., Foster City, CA 94404, USA; tinaiunsanlam@gmail.com

**Keywords:** monoclonal antibodies, mouse models, botulinum neurotoxins, toxin neutralization, toxicokinetics

## Abstract

Botulinum neurotoxins (BoNT) are some of nature’s most potent toxins. Due to potential food contamination, and bioterrorism concerns, the development of detection reagents, therapeutics and countermeasures are of urgent interest. Recently, we have developed a sensitive electrochemiluminescent (ECL) immunoassay for BoNT/B, using monoclonal antibodies (mAbs) MCS6-27 and anti-BoNT/B rabbit polyclonal antibodies as the capture and detector. The ECL assay detected as little as 1 pg/mL BoNT/B in the buffer matrix, surpassing the detection sensitivities of the gold standard mouse bioassays. The ECL assay also allowed detection of BoNT/B in sera matrices of up to 100% sera with negligible matrix effects. This highly-sensitive assay allowed the determination of the biological half-lives of BoNT/B holotoxin *in vivo*. We further tested the toxin neutralization potential of our monoclonal antibodies using the mouse systemic and oral intoxication models. A combination of mAbs protected mice in both pre- and post-exposure models to lethal doses of BoNT/B. MAbs were capable of increasing survival of animals when administered even 10 h post-intoxication in an oral model, suggesting a likely time for BoNT/B complexes to reach the blood stream. More sensitive detection assays and treatments against BoNT intoxication will greatly enhance efforts to combat botulism.

## 1. Introduction

Botulinum neurotoxins (BoNT) are some of the most potent natural toxins [1,2,3]. BoNTs are 150-kDa toxins produced by *Clostridium botulinum*, *C. butyricum*, and *C. baratii* [4,5,6]. BoNTs consists of a 100-kDa heavy chain (Hc) and a 50-kDa light chain (Lc). The 50-kDa Lc contains the endopeptidase catalytic domain that targets the soluble *N*-ethylmaleimide sensitive factor attachment protein receptor (SNARE) proteins and vesicle-associated membrane protein (VAMP/synaptobrevin) [3,7]. For example, synaptobrevin-II is the target for BoNT/B. Cleavage of either SNARE or VAMP proteins results in the inhibition of neurotransmitter release, leading to paralysis. The BoNT Hc contains the receptor binding and translocation domains that allow the toxin to be taken up by neurons [8,9]. BoNT holotoxins associate with neurotoxin-associated proteins (NAP) to form large toxin complexes. NAPs have been shown to provide protection from digestion and help promote BoNT intestinal binding and internalization [10,11].

Intoxication with BoNTs, either through ingestion of contaminated food, injection drug use, or through the environment, leads to the illness called botulism. In severe botulism cases, patients often require intensive care with mechanical ventilation. The therapeutics available are equine antitoxins and the Botulinum immunoglobulin (BabyBIG^®^) human antibody for use in infant botulism cases [1,12,13]. However, equine antitoxins could cause serious side effects, such as serum sickness and anaphylaxis; and the availability of BabyBIG^®^ is limited [14,15]. Neutralizing antibodies must also be administered early on during the course of intoxication, presumably before toxin is absorbed onto neurons, to be effective. Thus, there is a strong need for better and faster detection methods, and renewable and safe sources of neutralizing antibodies against BoNTs.

In order to accurately diagnose botulism, many tests on biological samples are performed. The gold standard for the detection of BoNTs is the mouse bioassay. The output for this assay is the death of at least two animals administered toxins, and the BoNT serotype involved is subsequently identified with antibody neutralization [16,17]. However, animal bioassays take at least 72 h to perform and require the use of animals. Therefore, several detection assays, usually immunoassay-based, are performed for quick confirmatory purposes. We have previously described the development of high affinity monoclonal antibodies (mAbs), MCS6-27 (against the Hc) and BoB92-32-1-10 (BoB92-32 in short, against the Hc [18,19,20,21]. These mAbs were used successfully in electrochemiluminescence (ECL) detection assays for BoNT/B in complex food matrices with limits of detection of 13 pg/mL [20]. Using these very sensitive methods, one can determine the toxicokinetics of BoNT *in vivo* [22].

In this study, we report the further optimization of the ECL assay using rabbit polyclonal anti-BoNT/B antibodies as the secondary detector. Using this improved method, the toxin levels in mouse sera over time were determined and compared with the timing of mAb rescue post-intoxication. We also determined the *in vivo* neutralization potential of single and combinatorial BoNT/B mAbs in systemic and oral models of botulism. The effects of antibody dosage and the timing of neutralizing antibody administration were tested. Increased knowledge of the *in vivo* half-lives of toxins, improved detection methods, and the identification of efficacious neutralizing antibodies will help advance treatments for botulism.

## 2. Results and Discussion

### 2.1. Detection of BoNT/B Using Electrochemiluminescent (ECL) Immunoassay

The gold standard for detection of BoNTs employs the mouse bioassay. The mouse bioassay can detect BoNT/B levels of 25 pg/mL [13,20,23]. However, these assays require about 3–4 days for full confirmation. To improve detection sensitivity and speed, we have previously described the development of high affinity monoclonal antibodies (mAbs), MCS6-27 and BoB92-32, and their use in ELISA detection of BoNT/B [19,21]. Both of these mAbs were against the Hc receptor binding domain (E859-E1291) of BoNT/B and were used successfully in electrochemiluminescence (ECL) detection assays in complex food matrices and horse sera [20]. Limits of detection for BoNT/B in buffer conditions were as low as 13 pg/mL. The ECL assays, like ELISA type immunoassays, take about 4–5 h to complete, but are less sensitive to food matrix effects. In addition, less sample volume is needed (15 μL *vs.* 50–100 μL) than an ELISA or animal bioassay.

**Figure 1:**
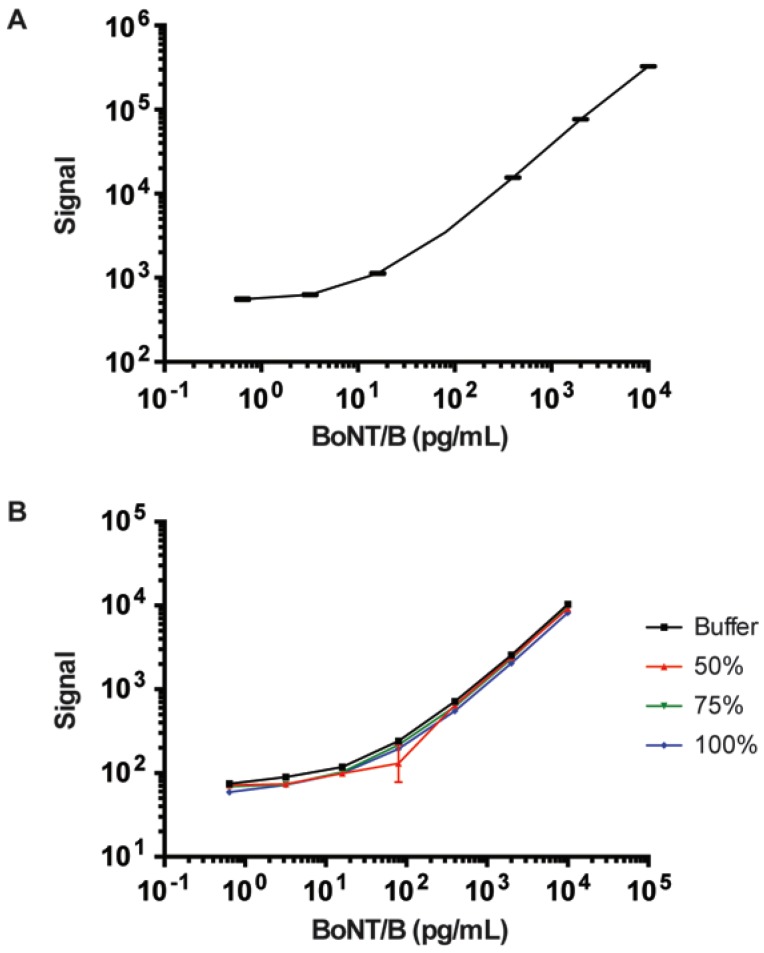
Electrochemiluminescent detection of BoNT/B with a MSD instrument. (**A**) Diagram of serial 1:5 dilutions of BoNT/B with a range of 10,000 to 0.64 pg/mL detected using an ECL assay using anti-BoNT/B mAb MCS6-27 for capture, and SULFO-TAG-labeled rabbit anti-BoNT/B polyclonal antibody for detection; and (**B**) the detection of BoNT/B dilution standards in the presence of buffer only or 50%, 75%, or 100% sera were compared. Graph points showed the mean ± SEM of duplicate wells.

Mice are highly sensitive to BoNT toxins. The LD_50_ for BoNT/B is about 12.5 pg for a 20 g mouse [20]. In order to determine the biologic half-life of BoNT/B holotoxins in mice, assays need to be able to detect low picogram amounts of BoNT/B in complex matrices, such as sera. To improve the sensitivity of the ECL assay, we tested the use of a rabbit polyclonal anti-BoNT/B antibody coupled with goat anti-Rabbit detector (SULFO-TAG labeled). We improved the limit of detection (LOD) for BoNT/B to 1 ± 0.1 pg/mL with a dynamic range for standard detection from 0.5 pg/mL to 100 ng/mL in buffer conditions (Figure 1A). Using this assay, we also tested the effect of fresh mouse sera matrix on detection sensitivity. Use of 50%, 75%, or 100% sera had negligible effects on detection sensitivity compared to the buffer matrix (Figure 1B). We believe the polyclonal rabbit antibodies contained multiple antibodies binding to different epitopes of captured BoNT/B; this, in turn, improved detection sensitivity. An improvement on detection sensitivity was also observed when multiple mAbs were used as detector antibodies in ELISA assays (data not shown).

**Figure 2:**
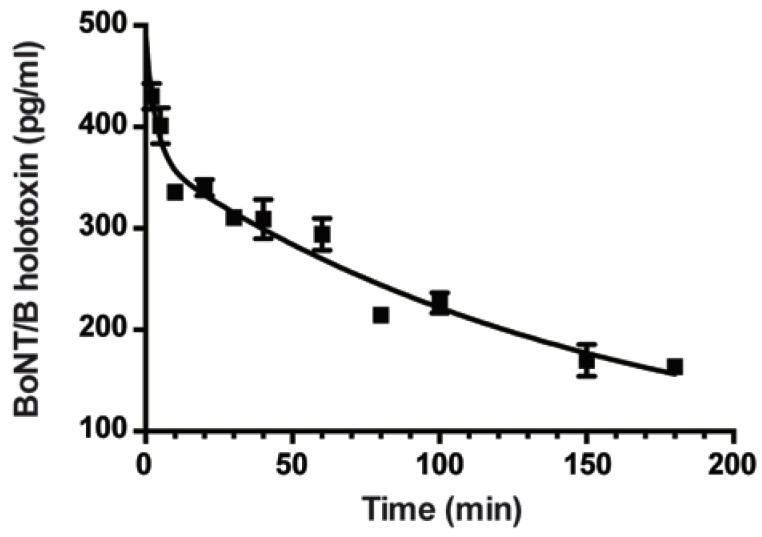
*In vivo* biological half-live of BoNT/B. Groups of five mice were injected with 1000 pg/mouse of BoNT/B and sera were obtained at 5, 10, 20, 30, 40, 80, 120, and 160 min post-intoxication. The concentration of unknown BoNT/B was determined using the ECL method. Each data point in graph represents the mean ± S.E.M. *R*^2^ = 0.8937.

We used this sensitive ECL assay to determine the biological half-lives of BoNT/B after intravenous (IV) introduction of toxin. Random sets of five mice were treated with 1000 pg BoNT/B holotoxin (about 80 mouse LD_50_) via tail vein IV injection. Sera were collected from each set of mice over time and the levels of BoNT/B were determined using the Meso Scale Discovery (MSD) instrument. Sera concentrations of BoNT/B over 3 h were then plotted (Figure 2). Soon after injection, BoNT/B holotoxin levels declined rapidly within the first 10 min of toxin introduction followed by a slower rate of toxin decline in the bloodstream. This initial phase or alpha half-life (*t*_1/2_α) was determined to be 2.3 min and represented the period of toxin redistribution to tissues, extracellular spaces and uptake by neurons (Figure 2). The second phase or *t*_1/2_β half-life was determined to be 105 min and likely represented the natural clearance of BoNT from the blood.

The serum half-life for BoNT/B was comparably faster than to those measured for BoNT/B in rats using radiolabeling [24]. This could be due to differences in toxin uptake in these two animals. Rats are not as susceptible to BoNT/B intoxication as mice due to differences in toxin receptors [25,26].

### 2.2. Neutralization of BoNT/B with Monoclonal Antibodies

**Figure 3:**
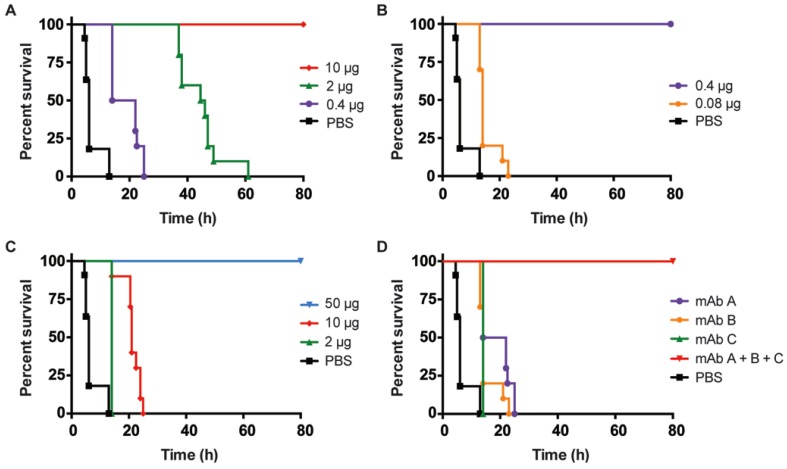
Monoclonal antibody neutralization of BoNT/B in the intravenous mouse model. The percent survival of mice treated with different doses of MCS6-27 (**A**); BoB92-23 (**B**); BoB92-32 (**C**); or Combo mAbs, containing 0.4 µg MCS6-27, 0.08 µg BoB92-23, and 2 µg BoB92-32 mAbs (**D**) one hour prior to challenge with BoNT/B were plotted over time. The percent survival of individual mAbs present in the Combo was also plotted for comparison. Control mice were treated with PBS iv instead of mAbs before intoxication.

We tested the neutralization potencies of individual and combinations of mAbs against BoNT/B in both the IV and oral mouse models of botulism. Three mAbs, MCS6-27, BoB92-23, and BoB92-32, all specific against the Hc receptor binding domain (amino acids E859-E1291) of BoNT/B were used alone or in combination to treat mice dosed with lethal doses of BoNT/B. These mAbs were first chosen because of their binding activity in BoNT/B capture assays. First, mice were administered different doses of mAbs by IV 30 min before injection with 1000 pg/mouse or about 80 mouse IV LD_50_ of BoNT holotoxin. Mice pre-treated with 10 µg of MCS6-27 or 0.4 µg BoB92-23, or 50 µg BoB92-32 prior to BoNT/B injection, were completely protected from death (Figure 3A–C). Control mice treated with PBS alone died with a median survival time of 6 h. Treatment with 0.4 µg MCS6-27, 0.08 µg BoB92-23, or 2 µg BoB92-32 only led to a slight delay in median survival time to 18, 14, and 14 h, respectively (Figure 3A–C). In contrast, treatment with 2.5 µg of a combination of these same concentrations of mAbs (from here referred as Combo mAbs: 0.4 µg MCS6-27, 0.08 µg BoB92-23, and 2 µg BoB92-32) conferred complete protection from death (Figure 3D).

Neutralization of BoNT/B with high doses of individual mAbs (such as 10 µg MCS6-27, 0.4 µg of BoB92-23, and 50 µg of 92–32) can fully protect mice from toxin lethality, but as reported by others, a combination of mAbs against different binding sites of BoNT had a synergistic effect on neutralization potential [27]. A combination of three single mAbs (Figure 3D) at much lower mAb concentrations can protect better, presumably because mAb binding at multiple toxin sites enables steric hindrance of receptor binding and also presumably by enhancing Fc domain binding and eventually immune clearance of toxins from blood. Previous studies have found that most BoNTs were cleared into the liver and not found associated with non-immune organs [24,28].

**Figure 4:**
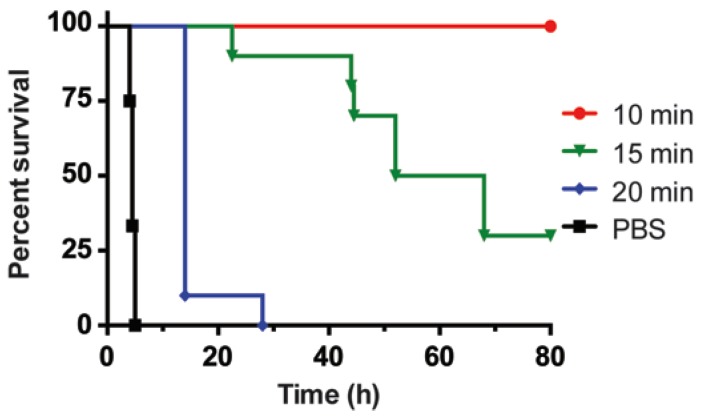
Neutralization of BoNT/B after intravenous intoxication. The percent survival of groups of 10 mice challenged iv with 1000 pg of BoNT/B holotoxin followed by treatment with PBS or 12.4 µg of Combo mAbs at 10, 15, and 20 min post-intoxication were plotted over time.

We wanted to determine the time frames when mAbs can rescue mice from death or delay the time-to-death. This information will help us understand whether there are windows of opportunity for treatment and would also give us clues to the timing of toxin passage in the mouse intestinal tract. Mice were treated IV with 1000 pg of BoNT/B holotoxin. A 12.4 µg dose of Combo mAbs (2 µg 6–27, 0.4 µg 92–23 and 10 µg 92–32) was then administered IV 5, 10, 15, 20, and 40 min post-intoxication. This dose, five times the dose used for complete protection of mice in Figure 3D, was chosen to ensure complete protection from intoxication. At this dose, toxin that is not neuron or tissue bound would be cleared from the system. Treatment with Combo mAbs 10 min or less after intoxication completely rescued mice from death (Figure 4). A time-to-death delay was seen when mice were given Combo mAbs 15 min post-intoxication with a median survival time of 60 h. Mice treated 20 min or more post-intoxication showed only a slight delay of time-to-death when compared with PBS-treated mice with median survival times of 14 and 4.5 h, respectively (Figure 4). This method helps predict the timing of when toxin is absorbed by neurons or tissues. At later time points, lethal amounts of BoNT/B would have been taken up and mAb neutralization would not prolong survival.

Both the half-life data and the post-IV intoxication mAb neutralization results indicate that once BoNT/B enters the blood stream, the window of time to neutralize toxins is short. The remaining BoNT/B that is not absorbed into neurons in the sera is slowly cleared. This remaining toxin reservoir is the target of current antitoxin therapies. For most foodborne cases of botulism, equine antitoxin therapy is recommended as early as possible after toxin confirmation. Positive treatment outcomes are directly correlated with early administration of antitoxins [1,14].

Compared to systemic intoxication, relatively large amounts of BoNT must be present to cause disease after oral ingestion [11,18]. Most of the BoNTs are likely degraded in the intestinal tract or shed as waste, with very little actually absorbed by the animal. Previous research showed that the BoNT/A complex was 17 times more toxic than BoNT/A holotoxin in the oral route of intoxication. This is likely due to protection from degradation of the botulinum toxin in the acidic environment and neurotoxin-associated proteins (NAP) mediated entry [10,11]. Although BoNT/A and BoNT/B complex have similar neurotoxin-associated protein (NAP) compositions, BoNT/B has been observed to form larger complexes than BoNT/A complexes in native gels [29]. BoNT/B complexes are also about 100× more toxic than BoNT/A complexes in oral intoxications of mice.

**Figure 5:**
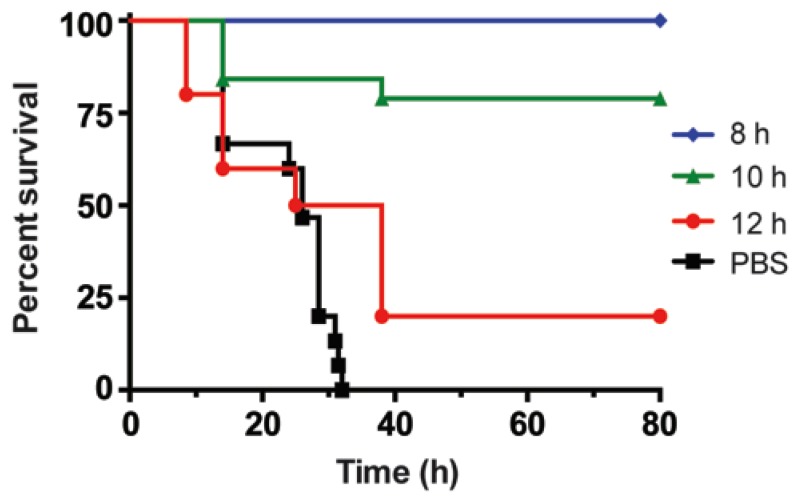
Neutralization of BoNT/B after oral intoxication. The percent survival of groups of 10 mice administered 0.06 µg or about three mouse oral LD_50_ of BoNT/B complex by gavage followed by rescue with 12.4 µg of Combo mAbs by IV at 8, 10, 12 h post-intoxication were plotted over time. Control mice were injected with PBS IV at 6 h post-intoxication instead of Combo mAbs.

In this study, we tested the neutralization of BoNT/B complex in the oral mouse model of intoxication. Mice were treated with 0.06 µg or about three oral LD_50_ of BoNT/B complex by gavage, followed by IV injection with 12.4 µg of Combo mAbs at (Figure 5). Intravenous injection of Combo mAb within 8 h conferred complete protection (defined as no lethality among treated mice). Treatment with the same dose of Combo mAb at 10 h post-intoxication reduced mortality to 20% and mice treated with Combo mAbs at 12 h had 80% lethality with a median survival of 32 h (Figure 5). Mice that survive toxin or antibody treatment will recover completely over time. Control mice treated with PBS at 6 h post-intoxication had a median survival of 26 h.

Orally-ingested BoNT/B would have to reach the blood stream or lymph to cause disease, thus one can predict the timing of when toxins reach those destinations by when protective antibodies cease to protect animals from intoxication. The timing of protection suggests that BoNT/B likely reaches the bloodstream at about 10 h after oral ingestion of BoNT/B complex. This is in sharp contrast to the much shorter oral protection window exhibited by mAb Combos against BoNT/A with BoNT/A complex transit through the intestinal tract and exit into the bloodstream estimated at about 7 h after oral ingestion [10,22,30]. The time between ingestion of toxin to the detection of BoNTs in the blood stream represented the time for toxin transit through the stomach, the intestinal tract, and receptor-mediated translocation through the intestinal epithelial cells into the blood stream and/or lymph [10]. Thus, it appears that the BoNT/B complex protected toxin better from intestinal degradation [31] but also slowed intestinal tract transit when compared with BoNT/A complex. It is not clear yet whether complex size or unique properties of the BoNT/B NAPs contributed to the increased toxicity of BoNT/B complex. Once orally-ingested BoNT reaches the blood stream, it would be quickly absorbed, following the systemic biologic half-life pattern (Figure 2). For both systemic and oral mouse antibody neutralization models, no mAb rescue was observed when mAbs were administered after visible symptoms of botulism (such as slowness, limping, *etc.*) became apparent.

### 2.3. Monoclonal Antibodies do not Inhibit BoNT/B Catalytic Activity

**Figure 6:**
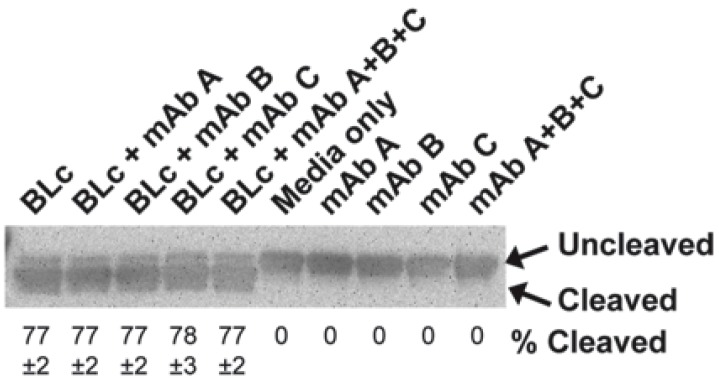
Individual or combination of mAbs does not inhibit BoNT/B endopeptidase activity. Endoprotease activity of the BoNT/B light chain (Lc) was measured by determining the percentage of cleavage of GstSynaptobrevin II after treatment with 2.5 µg of individual or Combo mAbs. Control reactions substituted Lc with buffer or by addition of buffer instead of mAbs. Percentage of cleaved GstSynaptobrevin II was determined for reactions with a visible cleavage fragment (mean ± S.E.M., *n* = 3).

MAbs can potentially neutralize BoNTs by blocking the endopeptidase activity of the BoNT Lc or by opsonization and clearance of antibody-toxin complex from the blood stream by immune cell processes [2,32,33,34]. Our mAbs bind to the Hc domain of BoNT/B and, thus, should not inhibit the catalytic activity of the toxin. However, this does not rule out that a combination of mAbs would not have such an effect. To test whether our individual or Combo mAbs-inhibited catalytic activity, we compared the cleavage of a recombinant GstSynaptobrevin II protein in the presence or absence of mAbs and the BoNT/B Lc. BoNT/B Lc cleaved the C-terminal of GstSynaptobrevin II substrate to yield a smaller protein that is visible in Western blots visualized with a HRP-labeled anti-Gst antibody. Control reactions with no mAbs present yielded a 77% cleavage of the full length protein (Figure 6). Addition of individual mAbs MCS6-27, BoB92-23, or BoB92-32, or the Combo mAbs did not inhibit the endopeptidase activity of BoNT/B Lc. In the absence of BoNT/B Lc, the presence of mAbs did not yield any cleavage of the GstSynaptobrevin II. This result suggests that the Combo mAbs are likely blocking toxin neuronal entry via steric hindrance, or by toxin clearance via opsonization through the liver or other immune organs, although, we cannot rule out that binding of three mAbs could cause a rare conformation change that leads to Lc activity inhibition.

## 3. Experimental Section

### 3.1. Reagents

Purified holotoxin and complex BoNT/B (Okra strain) were purchased from Metabiologics Inc. (Madison, WI, USA) and stored at 4 °C and −20 °C, respectively. Chemicals and reagents were generally purchased from Fisher Scientific (Walthe, MA, USA) or Sigma-Aldrich (Saint Louis, MO, USA) unless otherwise indicated. Toxin samples were diluted 1:100 in phosphate-gelatin buffer (0.028 M sodium phosphate, pH 6.2, 0.2% gelatin) and stored at −80 °C before use. Rabbit anti-BoNT/B antibody was purchased from Metabiologics and stored at 4 °C. Gst-Synaptobrevin-II substrate and BoNT/B light chain were purchased from List Biological Laboratories (Campbell, CA, USA). MAbs used were MCS6-27, BoB92-23-23-7 (or BoB92-23 in short), and BoB92-32-1-10 (or BoB92-32 in short) [19,20,21] toxins. Antibodies were purified from mouse ascite fluids by protein G-affinity chromatography. Purified antibodies were diluted in PBS, pH 7.4 and stored at −80 °C until use. Female Swiss Webster, 4–5 week old, mice were purchased from Charles River Laboratories (Portage, MI, USA).

### 3.2. Electrochemiluminescence (ECL) Immunoassay for the Detection of BoNT/B

MA2400 96-Well standard ECL plates (Meso Scale Discovery or MSD, Gaitherburg, MD, USA) were treated with 30 µL/well of a 2 µg/mL solution of anti-BoNT/B specific mAb MCS-6-27 in PBS and 0.05 M carbonate-bicarbonate buffer pH 9.6 and stored in 4 °C until use. Plates were blocked with 200 µL/well of Tris-Buffered Saline pH 8.0 (TBS: 0.05 M Tris, 0.138 M NaCl, 0.027 M KCl), 0.05% Tween-20 and 3% nonfat milk (TBS-T-NFM) for 1 h with shaking at 37 °C. Next, 30 µL/well of BoNT/B standards (ranging from 10,000 to 0.64 pg/mL) in TBS-T-NFM as well as serum samples (diluted 50% serum to buffer), were added and the microassay plates incubated at 37 °C with shaking for 1 h. Plates were then washed 3× with TBS-T buffer. For BoNT/B detection, 30 µL/well of a 2 µg/mL solution of biotinylated BoB92-32 was added to the wells. The plates were then incubated at 37 °C for 1 h, washed 3× in TBS-T µL. The plates were then incubated at 37 °C for 1 h, washed 3× in TBS-T. A 30 µL/well of a 1:1000 dilution of a ruthenium-conjugated SULFO-TAG™ Streptavidin (MSD) was then added and the plates incubated for 1 h at 37 °C with shaking followed with 3× washes with TBS-T buffer and the addition of 200 µL/well of 1× Read Buffer T with surfactant (MSD) before reading with a Sector Imager 2400 (MSD). Rabbit anti-BoNT/B polyclonal antibodies were also used instead of BoB92-32. Following incubation with 2 µg/mL rabbit antibodies, the assay plate is then incubated with a biotinylated goat anti-rabbit antibody, followed by addition of a ruthenium-conjugated SULFO-TAG™ Streptavidin. The amount of toxin present in sera was determined by comparison to the toxin standards in TBS-T-NFM included on each plate and by comparing the unknown signal to that of the buffer standard using the MSD Discovery Workbench software program (MSD). Each plate contained standards and samples in duplicate wells. The statistical significance of the ECL assays was determined using the Prism 6 program. LOD was determined by with standard equations in the Discovery Workbench software program. The LODs were reported as mean plus the standard error of the mean (SEM, *n* ≥ 4). Results from a typical standard curve for BoNT/B is shown in Figure 1A.

### 3.3. Mouse Intoxication and Neutralization Models

For the mouse systemic model, random groups of at least 10 mice were injected intravenously (IV) into the lateral tail vein with 100 µL of 10,000 pg/mL of BoNT/B holotoxin in phosphate gelatin buffer. 1000 pg/mouse is equivalent to about 80 intraperitoneal (IP) mouse LD_50_. In the oral model, 10 mice were treated with 0.06 µg each, equivalent to three mouse oral LD_50_, in 100 µL BoNT/B complex diluted in phosphate gelatin buffer via gavage using round-ended Popper needles. MAbs were diluted in sterile PBS, 100 µL of antibody solution were introduced iv into mice, either prior to or post-intoxication at indicated times. Surviving mice were monitored for at least 10 days for symptoms of intoxication or death. Health statuses of mice were observed over a 21-day period. All animal use protocols described here were approved by the Animal Care and Use Committee of the USDA, Western Regional Research Center, Albany, CA, USA.

### 3.4. Assay for BoNT Endopeptidase Activity

The cleavage of the recombinant GST-Synaptobrevin-II protein substrate by the BoNT/B light chain domain was performed as suggested by the manufacturer (List Biological Laboratories Inc., Campbell, CA, USA) with minor modifications. Briefly, 10 µM of GST-Synaptobrevin-II protein substrate was incubated with or without 60 nM of BoNT/B light chain and were then were treated with 2.5 µg individual mAbs or a combination of antibodies for 3 h at 37 °C (control samples were not pre-treated with mAbs) in a 10 µL reaction (20 mM HEPES, pH 7.4, 1.25 mM DTT, 0.3 mM ZnCl_2_ and 0.2% Tween-20). The reaction was then stopped by the addition of 4× LDS buffer (Invitrogen, Carlsbad, CA, USA) to give a final concentration of 1×, followed by heat inactivation (90 °C for 10 min). Products were then separated on SDS polyacrylamide gel electrophoresis (PAGE) using NuPAGE, 10% Bis-Tris gels (Invitrogen) followed by Coomassie blue staining or Western blotting. For Western blots, cleaved or un-cleaved recombinant GST-Synaptobrevin-II protein substrate was visualized by staining with a primary goat anti-Gst antibody (GE Healthcare city, Pittsburg, PA, USA) diluted 1:5000 in TBS-T and 5% milk buffer, followed by a secondary rabbit anti-Goat IgG peroxidase conjugated (Pierce). Stained gels and Western blots were analyzed with a FlurChem SP AlphaImager (Alpha Inotech, San Leandro, CA, USA). Molecular weight standards were purchased from Invitrogen. Statistical significance of data collected from five separate experiments was determined with unpaired *t*-tests using the Prism 6 statistics software (GraphPad Software Inc., San Diego, CA, USA).

## 4. Conclusions

This study aims to address two important areas of interest in the treatment of botulism: sensitive and timely detection of BoNTs and antibody-based therapies. As shown in the mouse models of systemic and oral toxin challenges and actual botulism cases, early delivery of neutralizing antibodies is imperative for rapid recovery from disease and decreased hospitalization times. We have developed an improved ECL assay for BoNT/B that has detection sensitivity of 1 pg/mL, more sensitive, less time consuming, and required less sample volumes than the current standard mouse bioassays. We have also tested the use of monoclonal antibodies in the neutralization of BoNT/B after both systemic and oral challenges in the mouse model. Small amounts of a combination of these mAbs protected mice from botulism. These antibodies could, in the future, be humanized and used as therapeutic antibodies. Currently, humanized anti-BoNT antibodies are being tested for use as therapeutics [35,36] and human antibodies purified from BoNT-immunized volunteers have been used successfully to combat botulism in infants [14,15]. Increasingly sensitive methods for toxin detection will allow quick diagnosis of disease and better knowledge and availability of BoNT neutralizing antibodies will help move us toward the design of more efficacious treatment for botulism.
